# Molecular epidemiology and antibiotic resistance of group B *Streptococcus* in pregnant women and neonates from Haikou, China: implications for vaccine development and antimicrobial stewardship

**DOI:** 10.3389/fcimb.2025.1655649

**Published:** 2025-09-09

**Authors:** Wenhui Mai, Huiting Wang, Qiaoyi Meng, Jingyi Zhang, Xinyi Gong, Zhulin Zhuo, Jinlei Sui, Xiaowen He, Yan Wang, Juan Li, Jianping Xu, Jinyan Wu

**Affiliations:** ^1^ Laboratory Department, Haikou Maternal and Child Health Hospital, Haikou, Hainan, China; ^2^ Key Laboratory of Tropical Translational Medicine of Ministry of Education, School of Basic Medicine and Life Sciences, Public Research Center, Hainan Medical University, Haikou, Hainan, China; ^3^ National Key Laboratory of Intelligent Tracking and Forecasting for Infectious Disease, National Institute for Communicable Disease Control and Prevention, Chinese Center for Disease Control and Prevention, Beijing, China; ^4^ Department of Biology, McMaster University, Hamilton, ON, Canada

**Keywords:** group B streptococcus, molecular epidemiology, antibiotic resistance, virulence factors, whole-genome sequencing

## Abstract

Group B *Streptococcus* (GBS) is a major cause of pregnancy complication and neonatal morbidity and mortality worldwide, particularly in developing regions. Despite its clinical importance, data on the molecular epidemiology, antibiotic resistance, and virulence factors of GBS in tropical regions are scarce. This study provides the first comprehensive analysis of GBS strains from pregnant women and neonates in Haikou, a tropical city in China, via antibiotic susceptibility testing and whole-genome sequencing. Our results grouped the 138 strains of GBS into seven serotypes and 28 multilocus sequence types (STs). These STs belonged to six clonal complexes (CCs). High antibiotic resistance rates were observed for tetracycline (89.1%) and clindamycin (55.1%) and the commonly detected resistance genes included *mreA* (100%), *ermB* (52.9%) and *tetM* (41.3%). Each strain contained at least one Pili-island (PI) gene and the capsular polysaccharide antigen among the GBS isolates were variably associated with CCs. All strains carried virulence genes *cfb* and *cylE*, followed by *pavA* (99.3%), and *lmb* (66.7%) etc. Our analyses showed ST862 as a dominant and potentially zoonotic genotype in Haikou, China, with implications for both human and animal health. The high prevalence of tetracycline and clindamycin resistance underscores the need for judicious antibiotic use and the development of region-specific antibiotic treatment guidelines. The discovery of novel STs and broad distributions of several virulence factors provide valuable insights for future vaccine development and targeted interventions in this region.

## Introduction

Group B *Streptococcus* (GBS) is a common type of bacteria frequently referred to as β-hemolytic, Gram-positive coccus that typically appears in pairs or chains. GBS is an opportunistic human pathogen but can colonize the human genital tract and lower digestive tract as part of the normal microflora. Microbiome studies have indicated that imbalances in microbial flora can readily trigger infections at various sites within the body (Dhudasia et al., 2021). Pregnant women, particularly during the perinatal period, represent a special vulnerable population. Hormonal changes during pregnancy often lead to a diversity of physiological shifts, including changes in the vaginal microbiota (Li et al., 2020). For example, elevated estrogen levels during pregnancy cause *Lactobacillus* to dominate the vaginal microflora, disrupting the vaginal microbial community which can lead to GBS infection of the birth canal. This can lead to adverse pregnancy outcomes, maternal infections, premature birth, miscarriage, stillbirth, and a range of poor maternal and neonatal outcomes such as neonatal sepsis, meningitis, or pneumonia (Okike et al., 2014; Pidwill et al., 2018; Yadeta et al., 2018; HogenEsch et al., 2021; Yuan et al., 2021).

Lancefield defined two types of carbohydrate antigens in the GBS wall: the Group B antigen, which is common to all strains, and the specific capsular polysaccharide antigen (Cieslewicz et al., 2005). Based on serological reactions that target the polysaccharide capsule or multiplex PCR, GBS can be classified into 10 serotypes: Ia, Ib, II, III, IV, V, VI, VII, VIII, and IX. These serotypes exhibit different geographical distributions (Cieslewicz et al., 2005; Slotved et al., 2007; Yao et al., 2013). Previous studies have reported that serotypes Ia, Ib, II, III, and V are commonly involved in invasive GBS infections (López et al., 2018; Huebner et al., 2022; Gharabeigi et al., 2023). Multilocus sequence typing (MLST) has also been used for the classification of GBS strains. Research findings indicate the existence of over 2000 MLST sequence types (STs), and certain serotypes and/or STs are associated with specific disease phenotypes (Luan et al., 2005; Jones et al., 2006; Madrid et al., 2017).

Current clinical treatments for GBS infections primarily rely on antibiotics such as penicillin, ampicillin, vancomycin, levofloxacin, tetracycline, moxifloxacin, linezolid, tigecycline, clindamycin, and quinupristin/dalfopristin. Due to antibiotic misuse and inadequate GBS screening, GBS infections can be hard to treat or prone to being mistreated, leading to high rates of adverse pregnancy outcomes and neonatal mortality (Bianchi-Jassir et al., 2017; Seale et al., 2017; Dong et al., 2020; Gonçalves et al., 2022). To reduce prenatal infections and adverse pregnancy outcomes, there has been increasing emphasis on improving clinical practice, strengthening screening, conducting universal testing for GBS infection among pregnant women, and implementing preventive treatments for potential mother-child infections.

Accurate diagnosis of infectious disease agents and quantitative data on their susceptibilities to antimicrobial agents are crucial for effectively preventing and treating adverse pregnancy outcomes, maternal infections, premature births, miscarriages, stillbirths, and a range of poor maternal and neonatal outcomes caused by GBS. However, much remains unknown about the molecular characteristics, virulence factors, or antibiotic resistance mechanisms of GBS strains from pregnant women and neonates, especially in tropical and developing regions such as Hainan Island in southern China. Here we investigated the molecular characteristics and antibiotic resistance patterns of GBS samples collected from pregnant women and neonates at the Haikou Maternity and Child Health Hospital in Hainan Province, China. For these GBS strains, we obtained data on their antibiotic susceptibilities and whole-genome sequences. This research contributes to the global effort to combat GBS infections and informs the development of targeted interventions, including vaccine developments and region-specific treatment guidelines.

## Materials and methods

### Sample collection

A total of 138 GBS isolates were obtained from 122 pregnant women and 16 neonates at Haikou Maternity and Child Health Hospital in Hainan Province, in tropical China, between 2021 and 2022. The samples from pregnant women included 108 vaginal secretions, 13 cervical secretions, and one amniotic fluid sample. The neonatal samples included 12 sputum samples from the lower respiratory tract, three throat swabs, and one blood sample. Specimen collections were performed in accordance with standard clinical protocols. Each of the 138 samples represented a unique patient visit. The details are provided in **Supplementary Table S1**.

### Isolation and identification of strains

All suspected GBS strains were transferred to Columbia blood agar plates and GBS chromogenic plates. All GBS isolates were incubated at 35-37 °C and 5% CO_2_/95% air for 18–24 h. The initial identification was performed via Gram staining, the CAMP test, and colony morphology. For further confirmation, the VITEK 2 Compact System (BioMérieux, Marcy-l’Étoile, France) with a GP colorimetric identification card was utilized to determine the species of GBS. Quality control was maintained via the use of *E. casseliflavus* ATCC 700327 and *E. faecalis* ATCC 29212.

### Antibiotic susceptibility test

To evaluate antimicrobial susceptibility, an AST GP67 card was employed in conjunction with the BioMérieux VITEK 2 system, following the manufacturer’s guidelines. Quality control was ensured by using *E. casseliflavus* ATCC 700327 and *E. faecalis* ATCC 29212 as reference strains. Multi-drug resistance (MDR) was defined as resistance to three or more categories of antimicrobial agents simultaneously.

### Whole-genome sequencing

To obtain the whole-genome sequences of the 138 strains, pure cultures of GBS were first cultivated for 18–24 h on Colombian agar with 5% sheep blood. The genomic DNA of each strain was extracted via the Wizard Genomic DNA Purification Kit (Promega, United States). The purified DNA was then sent to the MIGIGENE company for gene library construction and whole-genome sequencing (WGS) on the Illumina HiSeq 2000 platform. The *de novo* genome was assembled from Illumina data via the SPAdes (v3.13.1) software.

### Serotype analysis

Serotype identification via whole-genome sequencing (WGS) was performed following Metcalf’s methodology (Metcalf et al., 2017). For isolates where WGS-based serotype determination was unsuccessful, serotypes were resolved using a multiplex PCR approach (Imperi et al., 2010). Strains that remained serotype-untypable by both WGS and multiplex PCR were subjected to annotation via RAST (Rapid Annotation using Subsystem Technology; https://rast.nmpdr.org/) to screen for the presence of conserved capsular polysaccharide (*cps*) genes (Cieslewicz et al., 2005).

### Multilocus sequence typing

The specific sequence types (STs) and clonal complexes (CCs) were determined based on the gene sequences of the seven housekeeping genes, namely, *adhP*, *pheS*, *atr*, *glnA*, *sdhA*, *glcK*, and *tkt* (Jones et al., 2003). The STs and CCs were determined or newly identified when the genome sequences were uploaded to the MLST database (https://pubmlst.org/). The MLST minimum spanning tree was constructed via Bionumerics software (Applied Maths, Belgium).

### Data analysis

The antibiotic resistance genes and virulence factors were determined based on comparisons with data at the Center for Genomic Epidemiology database (CGE) (http://www.genomicepidemiology.org), the Comprehensive Antibiotic Resistance Database (CARD) (https://card.mcmaster.ca), and the Virulence Factor Database (VFDB) (https://www.mgc.ac.cn/VFs/), with a similarity threshold of > 90% and a coverage threshold of 60% compared with the reference sequences in the databases. For serotype and virulence factor gene analyses, we followed the protocols described in a previous study (McGee et al., 2021).

Statistical analysis was performed with GraphPad Prism 9.50 software. Analyses of the prevalences of different antibiotic resistance genes and virulence genes between CCs were conducted via the Chi-square test. Statistical significance was defined as *P*<0.05.

## Results

### Strain characteristics

A total of 138 GBS strains were collected from 16 neonates and 122 pregnant women. The neonates’ ages ranged from 1 hour to 28 days, while the pregnant women were categorized into two age groups: 96 were between 20 and 34 years old, and 26 were between 35 and 41 years old. Among the neonates, 8 were identified as high risk, 6 were diagnosed with neonatal pneumonia, 1 was a premature birth, and 1 was diagnosed with neonatal septicemia. Among the pregnant women, 38 were under normal pregnancy supervision, and the remaining 84 experienced adverse pregnancy outcomes. Further details regarding the samples are provided in **Table 1** and **Supplementary Table S1**.

**Table 1:** Clinical information of the patients with GBS in this study.

Host classification	Host characteristics		Number of isolates
Age	1 h-28 days		16
	20–34 years old		96
	35–41 years old		26
Clinical diagnosis	Neonates (16)	High-risk neonate	8
		Neonatal pneumonia	6
		Premature	1
		Neonatal septicemia	1
	Pregnant women (122)	Normal pregnancy supervision	38
		Premature rupture of membrane	41
		Threatened abortion	19
		High-risk pregnancy supervision	12
		Premature labor	4
		False labor	4
		Oligohydramnios	1
		Placenta previa and hemorrhage	1
		Cord around neck	1
		Pregnancy with cervical insufficiency	1
Total			138

### Antimicrobial susceptibility test results for GBS

*In vitro* susceptibility testing of 138 strains of GBS to 10 different antibiotics revealed that all strains were sensitive to the following six drugs: penicillin, ampicillin, vancomycin, linezolid, tigecycline, and quinupristin/dalfopristin. However, varying degrees of resistance/intermediate susceptibility were observed against the remaining four drugs, with the highest resistance rate of 89.1% against tetracycline, followed by clindamycin (55.1%), moxifloxacin (7.2%), and levofloxacin (5.1%) (**Table 2**). We detected nine resistance patterns among the 138 strains: tetracycline-clindamycin resistance accounted for 43.6% (60/138), followed by tetracycline resistance alone at 42.0% (58/138), and six strains (4.3%) were MDR, as detailed in **Table 3**.

**Table 2:** Results of antibiotic sensitivity testing for GBS [n (%)].

Antibiotics	Sensitive (S)	Intermediate (I)	Resistant (R)	I+R (%)
Tetracycline	15	0	123	123 (89.1)
Clindamycin	62	0	76	76 (55.1)
Moxifloxacin	128	0	10	10 (7.2)
Levofloxacin	131	1	6	7 (5.1)

**Table 3:** Antimicrobial resistance and cross-resistance patterns.

Pattern	Antibiotics resistance pattern (Antibiogram)	Total (n=138)
1	TE	58 (42.0%)
2	CLI	4 (2.9%)
3	LEV	1 (0.7%)
4	TE-CLI	60 (43.6%)
5	CLI-MFX	6 (4.3%)
6	TE-CLI-LEV	2 (1.4%)
7	CLI-LEV-MFX	1 (0.7%)
8	TE-CLI-LEV-MFX	3 (2.2%)
9	NONE	3 (2.2%)

TE, tetracycline; CLI, clindamycin; LEV, levofloxacin; MFX, moxifloxacin.

### Distribution of GBS serotypes

**Figure 1:**
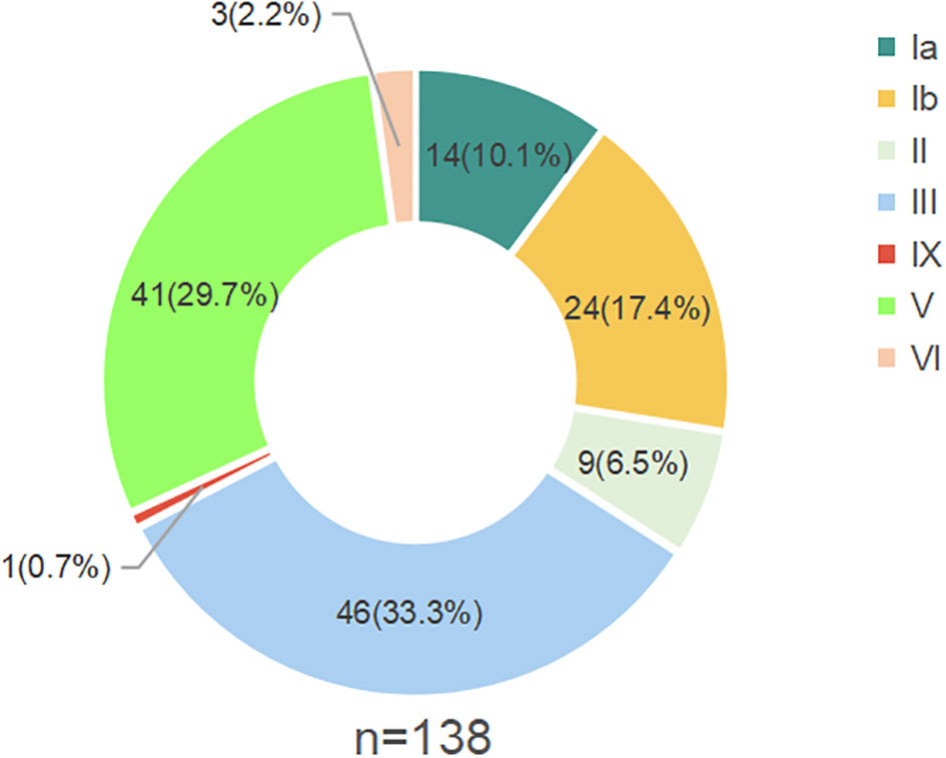
Capsular polysaccharide serotypes of the GBS strains; n: Total number of isolates.

Based on DNA sequences at genes involved in capsular polysaccharide (CPS) synthesis, the 138 GBS strains were classified into seven serotypes: Ia, Ib, II, III, V, IX and VI. The most prevalent serotype was III, representing 33.3% of the strains (46 out of 138), followed by serotype V, which accounted for 29.7% (41 strains). The remaining serotypes included Ib (17.4%), Ia (10.1%), II (6.5%), VI (2.2%), and IX (0.7%) (**Figure 1**).

### Multilocus sequence typing of GBS

**Figure 2:**
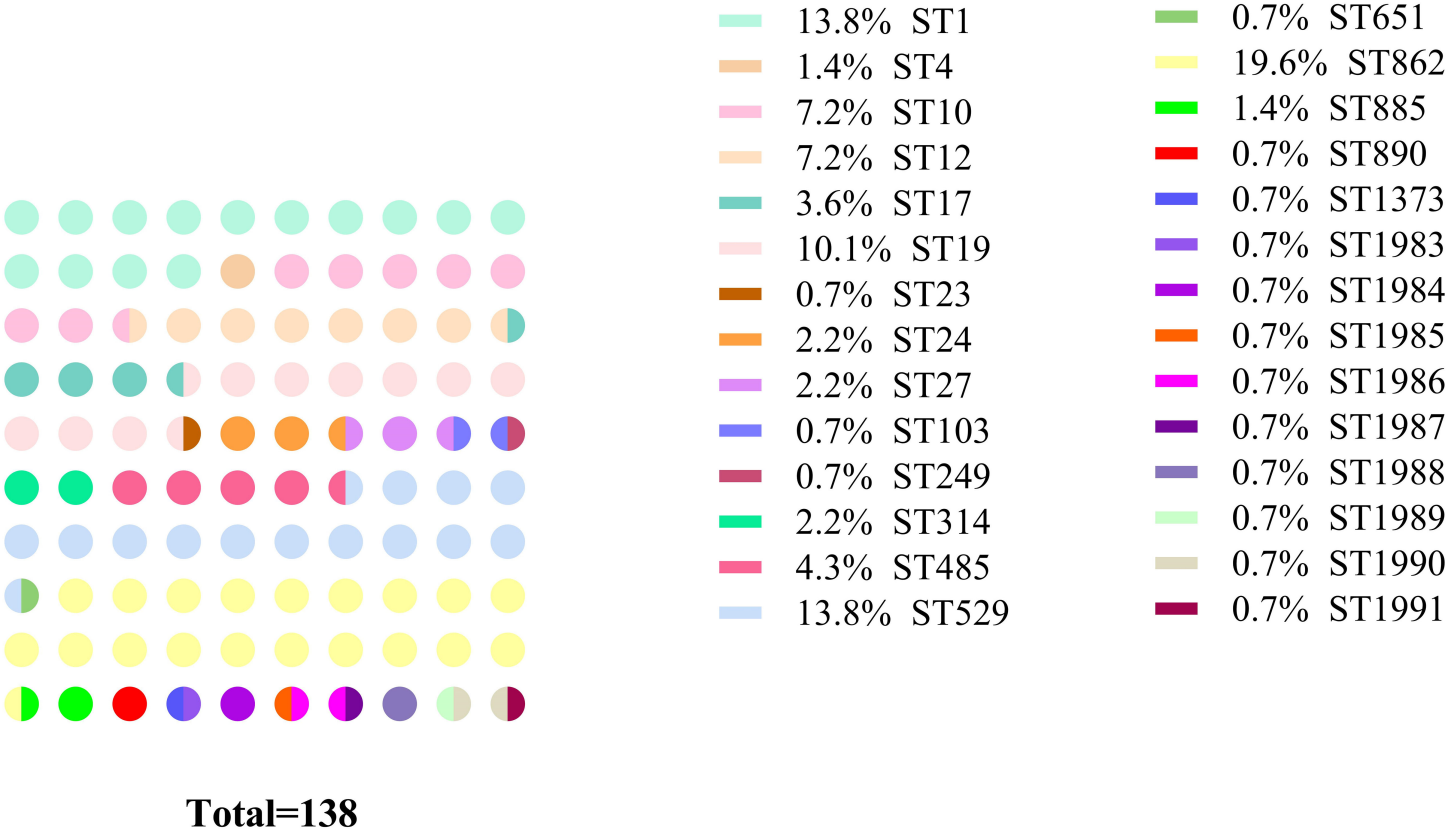
MLST results for the 138 GBS strains. The color blocks within circles indicate STs, and the number of circles indicates the proportion of the corresponding STs.

Based on comparisons with data in the MLST database for GBS at seven gene loci, the 138 GBS strains were identified as belonging to 28 STs, including nine new ones (ST1983–1991). Among these 28 STs, 13 STs were shared by a total of 123 strains, and the remaining 15 STs were each represented by one isolate. The top three most prevalent STs were ST862 (19.6%, 27/138), followed by ST529 (13.8%, 19/138) and ST1 (13.8%, 19/138). Each of the nine new STs was represented by only one strain each. The distributions for all 28 STs are detailed in **Figure 2**.

### Clonal complexes of the 138 GBS strains

**Figure 3:**
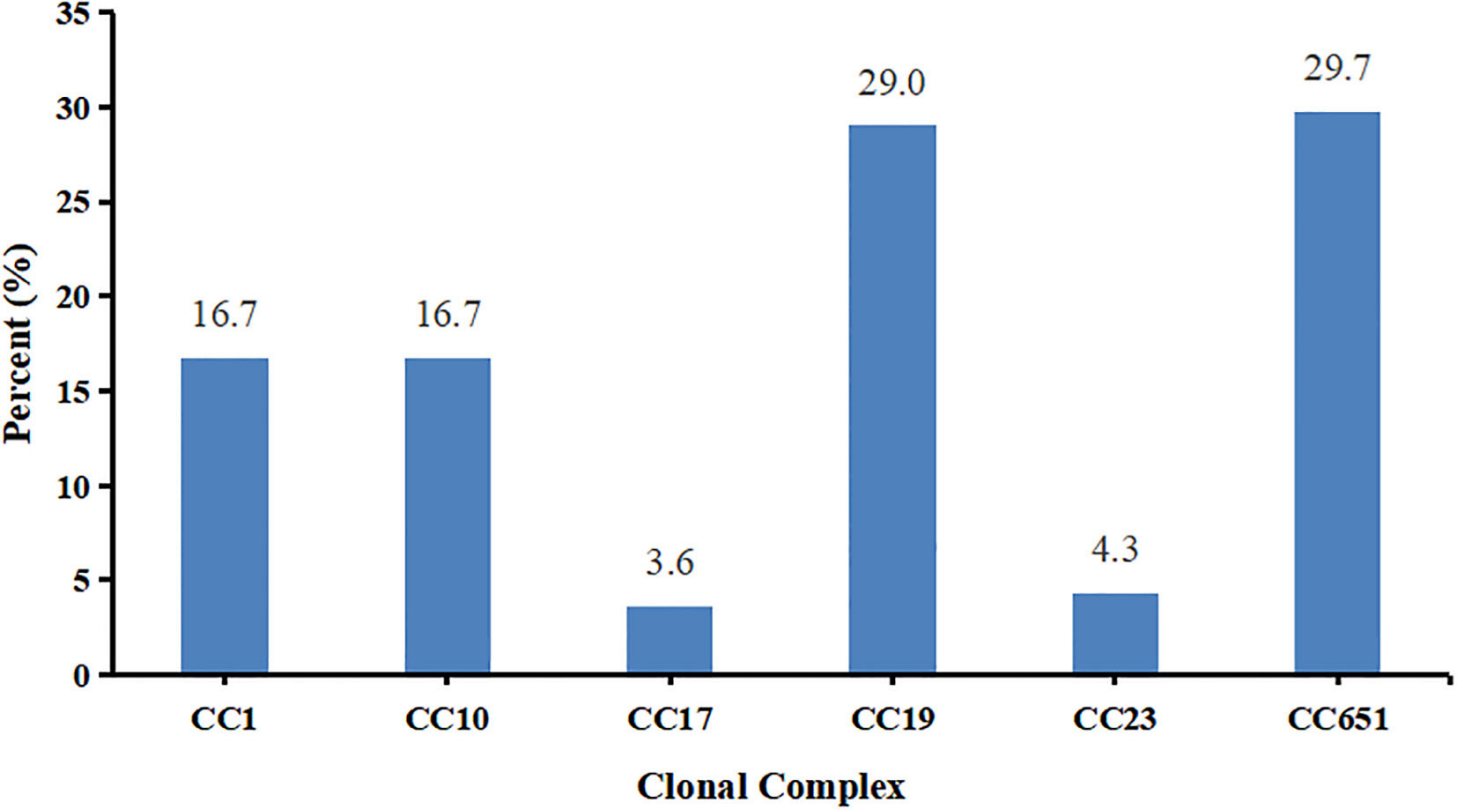
Distribution of clonal complexes of the 138 GBS strains in Haikou, China.

The 28 STs were grouped into 6 clonal complexes (CCs). The relative proportions of these CCs from the most frequent to the least frequent were CC651 with 41 strains (29.7%), CC19 with 40 strains (29.0%), CC1 and CC10 with 23 strains (16.7%), CC23 with 6 strains (4.3%), and CC17 with 5 strains (3.6%) (**Figure 3**).

**Figure 4:**
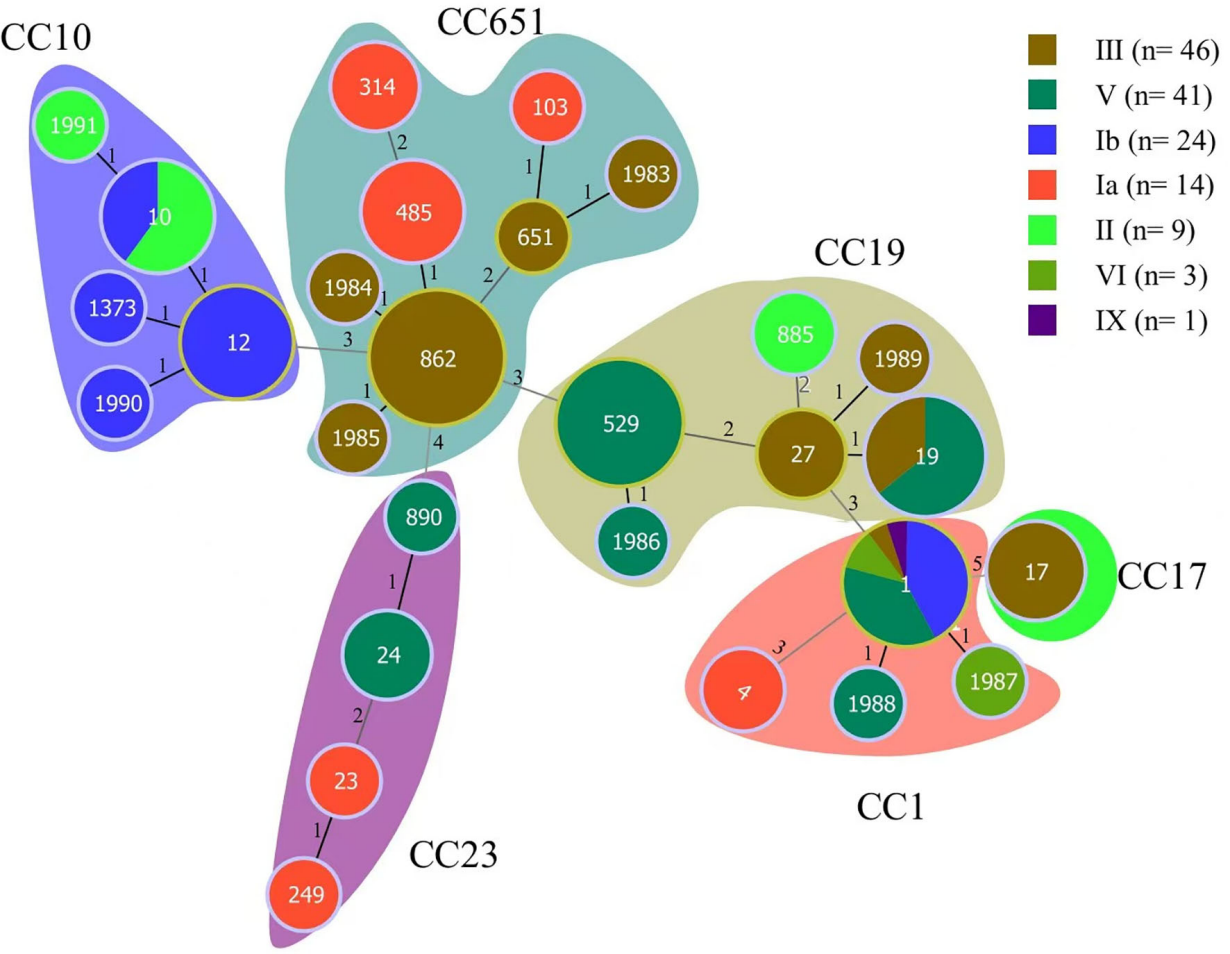
Distributions and relationships among multilocus sequence type, clonal complex, and serotype in the collection of 138 GBS isolates. Labels within individual circles indicate the STs, the sizes of circles are proportional to the number of isolates, and their colors represent serotypes. The variably shaped colored blocks indicate the clonal complex to which the STs belong. The STs from ST1983 to ST1991 were novel types detected in the study.

The 41 CC651 strains belonged to two serotypes III and Ia, with serotype III being more common than serotype Ia (*P*<0.001). The 40 CC19 strains belonged to three serotypes II, III, and V, with serotype V being more frequent than serotypes II and III (*P*<0.001). The 23 CC10 strains belonged to serotypes Ib and II, with serotype Ib being more frequent than serotype II (*P*<0.05). The 23 CC1 strains belonged to six serotypes, excluding serotype II. The six CC23 strains belonged to serotypes V and Ia and all 5 CC17 strains were classified as serotype III. The detailed data showing the relationships among multilocus sequence type, clonal complex, and serotypes are shown in **Figure 4** and **Supplementary Table S2**.

### Antibiotic resistance genes

**Figure 5:**
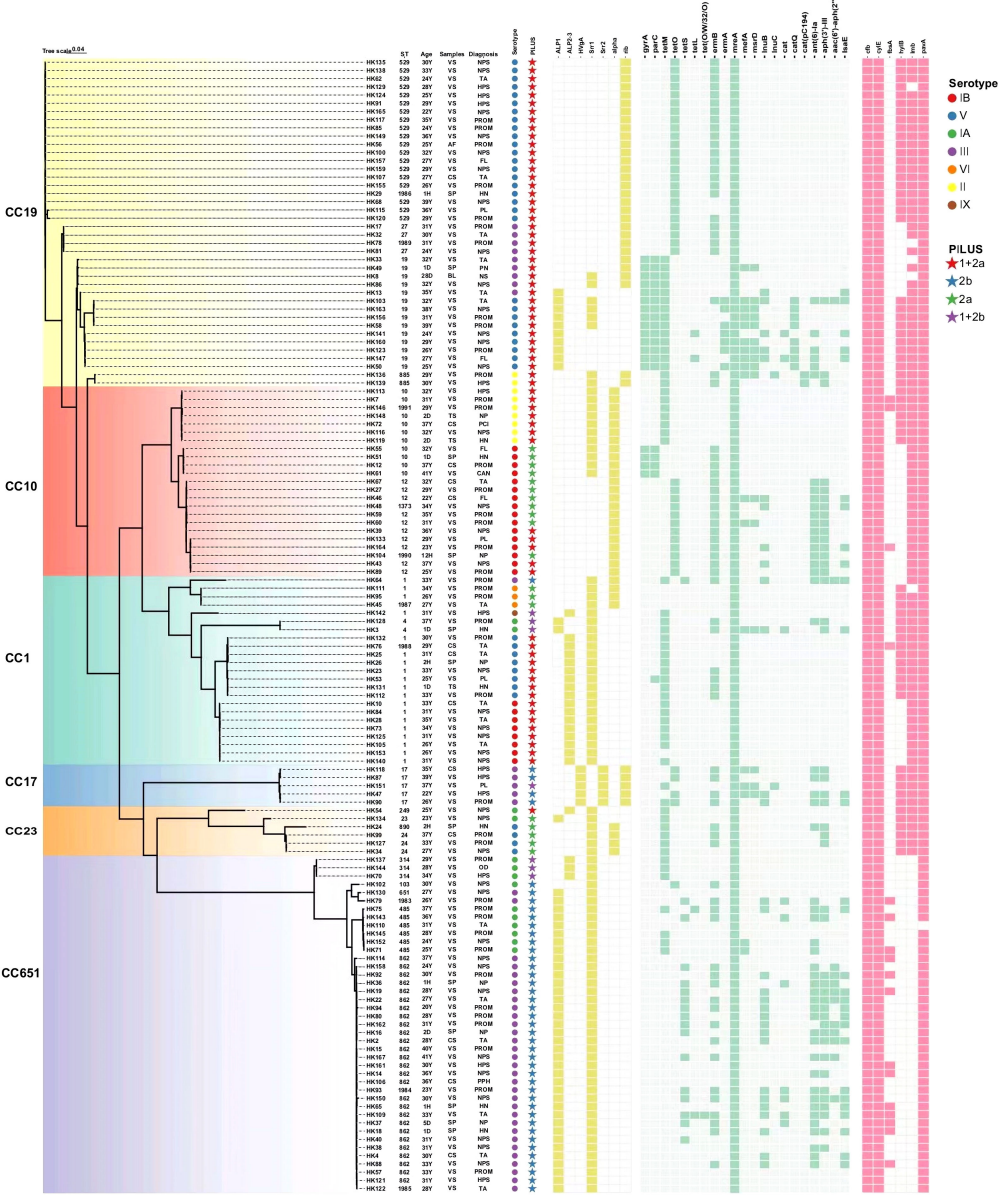
Carriage of antibiotic resistance and virulence genes in 138 strains of GBS from pregnant women and neonates. CS, cervical secretions; SP, sputum; BL, blood; TS, throat swabs; AF, amniotic fluid; VS, vaginal secretions. HN, high-risk neonate; NP, neonatal pneumonia; PN, premature neonate; NS, neonatal septicemia; NPS, normal pregnancy supervision; PROM, premature rupture of membrane; TA, threatened abortion; HPS, high-risk pregnancy supervision; PL, premature labor; FL, false labor; OD, oligohydramnios; PPH, placenta previa and hemorrhage; CAN, cord around neck; PCI, pregnancy with cervical insufficiency.

A total of 19 antibiotic resistance genes were identified across the 138 strains of GBS. These genes confer resistance to various classes of antibiotics, including macrolides, lincosamides, tetracyclines, chloramphenicols, and aminoglycosides. Specifically, the identified resistance genes included those resistant to (i) macrolide and lincosamide drugs such as *ermB*, *ermA*, *mreA*, *mefA*, *msrD*, *lunB*, *lsaC*, and *lsaE*; (ii) tetracycline such as *tetM*, *tetO*, *tetS*, *tetL*, and *tet(O/W/32/O)*; (iii) chloramphenicol such as *cat*, *catQ*, and *cat(pC194)*; and (iv) aminoglycoside such as *ant(6)-Ia*, *aph(3’)-III*, and *aac(6’)-aph(2’’)*. Notably, all 138 strains carried the *mreA* gene, with 52.9% carrying the *ermB* gene and 41.3% carrying the *tetM* gene. In addition, all strains in CC19 carried the *catQ* or the *cat(pC194)* genes. The resistance gene *tet(O/W/32/O)* was detected in only one strain. The resistance genes *Gyr*A and *Par*C were found in only 13.0% and 13.8% of the strains respectively, with these two genes being exclusively (*Gyr*A) or predominantly (*Par*C) found in CC10 and CC19 strains (**Figure 5**).

### Surface protein genes and virulence factors

In this study, virulence factors encompassing various cell surface proteins that facilitate GBS adhesion and invasion were identified. These virulence factors include members of the pilus islands (PI-1, PI-2a, PI-2b), alpha-like protein (Alp) family (Alpha C, Alp1, Alp2/3, Rib), and serine-rich repeat glycoproteins (Srr1 and Srr2). All strains were found to contain at least one Pili-island (PI) gene. The most prevalent combination was PI-1+PI-2a, which was found in 50.0% of the strains (69/138), followed by PI-2b (31.2%, 43/138), PI-2a (13.8%, 19/138), and PI-1+PI-2b (5.1%, 7/138). Among the 138 strains, all CC19 strains were found to carry the PI-1+PI-2a combination. The PI-2a was identified in strains belonging to CC1, CC10, and CC23. The PI-2b was identified in 92.7% of CC651 strains, a rate much higher than that in other clonal complexes (*P*<0.001). The PI-1+PI-2b was identified in strains belonging to CC1, CC17, and CC651. In contrast, only PI-2b was identified in all CC17 strains, with the exception of one ST17/CC17 strain that carried PI-1+PI-2b, as shown in **Figure 5**. The *Alp1* gene was found in 90.2% of the CC651 strains, which accounted for a significantly greater proportion than those in CC1, CC19, and CC23 (*P*<0.001). Conversely, the *Alp2/3* gene was detected in 73.9% of the CC1 strains, which was much higher than that reported for the other CCs (*P*<0.001). A total of 64.5% of the isolates in this study contained the *Srr1* gene. The strains carrying *HvgA* or *Srr2* were exclusively in CC17, with a 100% carrying rate. The *rib* gene was found in 75.0% of the CC19 strains, which was a significantly greater percentage than that in the CC17 strains (*P*<0.001), as illustrated in **Figure 5**.

The *cfb* and *cylE* genes were detected in all 138 strains. The *pavA* gene was almost universally present (99.3%, 137/138), while the detection rates for the *lmb*, *hylB*, and *fbsA* genes were 66.7%, 45.7%, and 12.3%, respectively.

### Distribution and characterization of GBS strains associated with pregnancy outcomes

In six neonatal pneumonia cases, ST862/CC651/serotype III was the predominant genotype, accounting for 50% (3/6) of the cases (**Table 4**). The only observed case of neonatal septicemia was caused by a strain belonging to ST19/CC19/serotype III. Notably, ST17/CC17/serotype III has been exclusively identified in strains associated with adverse pregnancy outcomes and the only strain harboring the *HVgA* gene (**Table 4**). Interestingly, the nine newly identified STs in this study were all associated with invasive diseases and adverse maternal outcomes (**Table 4**). However, aside from the above, there was no statistically significant difference in the distribution of CCs and serotypes between adverse pregnancy outcomes and normal pregnancies.

**Table 4:** Detailed clinical outcome and strain genotype information among the 138 strains of GBS.

Sample source	Clinical outcomes	Number of isolates	STs	CCs	Virulence gene	Surface protein gene and virulence factors	Serotypes
Neonates (16)	Neonatal septicemia	1	19	19	*cfb, cy1E, pavA*	PI-2a, Srr1, Rib	III
	Neonatal pneumonia	6	862^3^, 1, 10, 1990	651^3^, 10^2^, 1	*cfb* ^6^, *cy1E* ^6^, *pavA* ^6^, *1mb* ^3^, *hy1B* ^2^, *fbsA*	Srr1^5^, ALP1^3^, PI-2b^3^, PI-1+PI-2a^2^, ALP2-3, alpha, PI-2a	III^3^, IB, II, V
	High-risk neonate	9	862^2^, 10^2^, 1, 890, 4, 1986, 19	651^2^, 10^2^, 1^2^, 19^2^, 23	*cfb* ^9^, *cy1E* ^9^, *pavA* ^8^, *1mb* ^6^, *hy1B* ^5^, *fbsA* ^2^	Srr1^7^, PI-1+PI-2a^4^, ALP1^3^, alpha^3^, PI-2a^2^, PI-2b^2^, rib^2^, ALP2-3, PI-1+PI-2b	V^3^,III^3^, IA, IB, II,
Pregnant women (122)	Adverse outcomes	84	862^13^, 529^12^, 1^11^, 12^8^, 19^7^, 10^6^, 17^5^, 485^5^, 314^3^, 885^2^, 24^2^, 27^2^, 4, 1983, 1984, 1985, 1987, 1988, 1989, 1991	651^24^, 19^24^, 1^14^, 10^12^, **17** ^5^, 23^2^	*cfb* ^84^, *cy1E* ^84^, *pavA* ^83^, *1mb* ^56^, *hy1B* ^40^, fbsA^10^ ***HVgA*** ^5^	Srr1^52^, PI-1+PI-2a^39^, ALP1^27^, PI-2b^26^, rib^23^, alpha^21^, PI-2a^13^, ALP2-3^12^, PI-1+PI-2b^6^, **Srr2^5^**	III^27^, V^24^, 1B^14^, IA^9^, II^6^, VI^3^, IX
	Normal pregnancy	38	862^9^, 529^7^, 1^6^, 19^5^, 12^2^, 10, 23, 24, 27, 103, 249, 485, 651, 1373	19^13^, 651^12^, 1^6^, 10^4^, 23^3^	*cfb* ^38^, *cy1E* ^38^, *pavA* ^38^, *lmb* ^26^, *hy1B* ^16^, *fbsA* ^4^	Srr1^24^, PI1 + 2a^23^, ALP1^16^, PI-2b^12^, rib^9^, alpha^5^, ALP2-3^7^, PI-2a^3^	V^13^, III^12^, IB^7^, IA^4^, II

Superscript numbers represent the number of strains in this population with the specific gene, genotype, or serotype.

Bold texts refer to those found only in patients with adverse pregnancy outcomes.

## Discussion

GBS is a common pathogen that can cause adverse pregnancy outcomes and is a significant cause of neonatal sepsis and meningitis within the first 90 days of life. The integration of GBS screening into pre-pregnancy and prenatal care guidelines by the Obstetrics and Gynecology Branch of the Chinese Medical Association in 2014 underscores the importance of early detection and intervention. Our study provides critical insights into the molecular epidemiology of GBS in Haikou, China, revealing that serotypes Ia, Ib, II, III, and V collectively account for 97.1% of the cases. This distribution aligns with global patterns, highlighting the universality of these serotypes in GBS infections (Teatero et al., 2017; Furfaro et al., 2018). However, the most prevalent GBS serotypes vary among different regions. In our study, serotypes III (33.3%), V (29.7%), and Ib (17.4%) were more prevalent than the global averages of 25%, 18%, and 8%, respectively, while serotypes Ia (10.1%) and II (6.5%) were less prevalent than the global averages of 21% and 11%, respectively (Russell et al., 2017). In Beijing, China, serotype III predominated with a smaller proportion of serotype V (Wang et al., 2015), whereas in Rio de Janeiro, Brazil, serotype Ia was the most common, with serotype III being less prevalent (Costa et al., 2022). India reported a lower prevalence of serotype III (11%) and no serotype V. In Japan, serotypes III and V were less prevalent, whereas VI and VIII were the most prevalent serotypes. These regional differences underscore the necessity for localized surveillance to inform tailored prevention and treatment strategies. Continuous monitoring of serotype shifts is critical for guiding vaccine development and adjusting vaccine compositions to meet evolving prevention and control needs.

Globally, ST17, ST1, ST23, and ST19 are the predominant STs associated with GBS infections in pregnant women (Manning et al., 2008; Shabayek and Spellerberg, 2018). Notably, ST17 and ST19 were more prevalent in strains causing early-onset disease (EOD) and late-onset disease (LOD) respectively (Manning et al., 2009). A total of 28 STs were identified in this study, with ST862 being the most prevalent, followed by ST529 and ST1. Similar to serotype distribution patterns described above, the predominant sequence types vary among geographic areas. For example, previous studies showed that ST19 and ST10 were the most common STs of GBS in pregnant women in China, followed by ST12, ST17 and ST651 (Wang et al., 2023). In our study, ST862 emerged as a dominant sequence type in Haikou, similar to findings from Xiamen and Fuzhou cities in Fujian Province, although it has rarely been reported in other parts of China (Jiang et al., 2016; Wu et al., 2019; Yao et al., 2020; Liang et al., 2023). Importantly, ST862 is also an emerging zoonotic ST that potentially poses a threat to both human health and animal husbandry (Sapugahawatte et al., 2022). These findings suggest that ST862 may possess a high degree of adaptability and transmission capacity in these regions, possibly due to its specific biological characteristics, host preference, or environmental adaptability. Similarly, ST529, another prevalent ST in our study, has been reported as a major sequence type in Linyi, Shandong Province but is rarely observed in other regions (Zhou et al., 2024). Our findings highlight the importance of monitoring spatial and temporal distributions of STs to identify emerging STs, particularly those with zoonotic potential, to mitigate their impact on public health in this and other microbial pathogens (e.g., Dalmieda et al., 2024; Poorrashidi et al., 2024; Meng et al., 2025).

A study by Kimura et al. (2013) revealed that the sensitivity of GBS strains to penicillin is decreasing worldwide, and penicillin is still the first-line choice for the treatment of GBS infection because of its excellent therapeutic effect (Quiroga et al., 2008; Petca et al., 2024). In our study, no penicillin-resistant strains were observed, reinforcing the continued use of penicillin as the primary treatment for GBS infections. However, for patients with severe penicillin allergies, clindamycin is an important alternative. Erythromycin, on the other hand, is less effective because of its poor placental penetration, limiting its clinical use (Elbiss and Osman, 2023). Recent reports indicate that resistance to erythromycin and clindamycin is increasing (Sabroske et al., 2023), emphasizing the need for antibiotic selection based on drug sensitivity testing at regional and local levels. In our study, high resistance rates were observed for tetracycline (89.1%) and clindamycin (55.1%), suggesting that these drugs should not be used without prior susceptibility testing in patients with penicillin allergies in Haikou, China. Additionally, resistance rates for moxifloxacin (7.2%) and levofloxacin (5.1%) in our strains were higher than those reported in Taiwan (Tsai et al., 2022). Variations in antibiotic usage patterns likely explain the geographical differences in resistance prevalence. In summary, while penicillin is still preferred for GBS infections, the high resistance rates to alternative antibiotics, particularly tetracycline and clindamycin, necessitate careful antibiotic selection guided by sensitivity testing to optimize treatment outcomes and reduce resistance.

Tetracycline resistance in streptococci is mediated by ribosome protection genes such as *tet(M)* and *tet(O)* or by the efflux pump genes *tet(K)* and *tet(L)*. Macrolide resistance is due primarily to two mechanisms: ribosomal methylation encoded by the *erm* gene and active efflux mediated by a membrane-bound protein encoded by the *mef* gene. The former mechanism often confers high-level resistance to macrolides, lincosamides, and streptogramin B antibiotics. In our study, resistance to tetracycline was attributed to the presence of *tet(O)*, whereas erythromycin resistance was linked to the *ermB* gene, which encodes erythromycin ribosome methylase. Clindamycin resistance was associated with *lnuB*, a gene encoding a lincosamide-inactivating nucleotidyl transferase, and kanamycin resistance was linked to *aphA3*, encoding an aminoglycoside phosphotransferase. The average correlation between resistance phenotypes and resistance genes of GBS was 65.62%, with a 100% correlation for tetracycline resistance. Similar findings have been reported for GBS strains isolated from Brazilian mastitic cows (Silva et al., 2017) and *Staphylococcus aureus* strains isolated from raw milk (Liu et al., 2017). However, our study revealed that *lnuB* was detected in only 16 of 76 clindamycin-resistant strains, suggesting that clindamycin resistance may involve other yet unidentified mechanisms.

Consistent with previous studies (Springman et al., 2014; Liu et al., 2023), we observed that PI-1 and PI-2a are the most common PI family genes in GBS strains. In our study, PI-2a was present in 63.8% of the strains, followed by PI-1 (55.1%) and PI-2b (36.2%). Notably, the combination of PI genes was more prevalent than individual PI genes were, a finding supported by previous studies in China (Liu et al., 2023) and Ireland (Meehan et al., 2014). All the isolates in our study carried at least one PI gene, with the PI-1+PI-2a combination being the most common (50.0%). This result aligns with the findings of Khodaei et al (Khodaei et al., 2018). Interestingly, while previous studies suggested that the PI-1+PI-2b combination was exclusive to CC17 isolates, our study revealed that only PI-2b was present in all CC17 strains, except for one ST17/CC17 strain that carried PI-1+PI-2b.

The virulence factor *HvgA* is an adhesin found in highly virulent strains that are frequently associated with neonatal meningitis. *HvgA* enhances bacterial adhesion to intestinal epithelial cells, choroidal epithelial cells, and microvascular endothelial cells that constitute the blood-brain barrier (Choi et al., 2022). Similarly, the Rib protein has been implicated in promoting neonatal meningeal infection. These surface-anchored proteins not only facilitate bacterial adhesion but also enable GBS to penetrate the intestinal and blood-brain barriers, allowing migration to the circulatory and central nervous systems (Fischer et al., 2021). In our study, *HvgA* was predominantly associated with serotype III/CC17 strains, which is consistent with the findings of Bourrel et al (Bourrel et al., 2024), who demonstrated that *HvgA* enhances the adhesion of CC17 strains, making them more virulent than non-CC17 strains. This highlights the need for enhanced clinical monitoring of CC17 strains to better understand their epidemiology, infection characteristics, and resistance patterns, thereby informing prevention and control strategies.

Another surface adhesin, *Srr1*, was detected in all CCs except CC17, whereas *Srr2* was exclusively found in CC17 strains. This finding is consistent with studies by Lacasse et al. (2022) and Jin et al (Jin et al., 2022), who reported that *Srr2* was strongly associated with CC17 strains, particularly in cases of GBS infection in infants. These results suggest that *Srr2* may play a role in the virulence of CC17 strains, further emphasizing the importance of monitoring this clonal complex.

The virulence factors identified in this study, including *cfb*, *cylE*, *pavA*, *lmb*, and *hylB*, are potential candidates for vaccine development. All the strains carried *cfb* and *cylE*, while *pavA* (99.3%) and *lmb* (66.7%) were also highly prevalent. In contrast, *fbsA* was detected in only 12.3% of the strains, making it a less suitable candidate for vaccine epitopes. These findings provide valuable insights for the development of targeted vaccines against GBS.

Overall, our data indicate that multilocus sequence types ST862, ST529, ST1, and ST19 were the predominant GBS genotypes in this region. Additionally, we found nine novel STs (ST1983–1991) never reported before in other geographic regions, all of which were associated with adverse pregnancy outcomes. In this GBS population, the serotype distribution was dominated by III, V, 1b, and 1a and virulence factors such as Srr1, Srr2, ALP1, PI-1, PI-2a, PI-1, and PI-2b were closely associated with maternal infections. Nearly all strains carried virulence genes *cfb*, *cy1E*, and *pavA*. Together, these data provide valuable references for the development of targeted GBS vaccines in this population. A limitation of this study is the absence of longitudinal follow-up with pregnant women and their neonates, which restricts our understanding of maternal colonization by invasive GBS serotypes and subsequent vertical transmission to and potential effects on neonates. Additionally, due to the retrospective nature of this cohort study, we were unable to establish direct causal relationships between specific strains and adverse outcomes. Future studies incorporating longitudinal follow-up and prospective data collection could provide deeper insights into the epidemiology and pathogenesis of GBS infections in this vulnerable population, while mitigating limitations related to causal inference.

## Conclusions

This study highlights the emergence of ST862 as a dominant and potentially zoonotic sequence type of GBS in Haikou, China, with significant implications for public health and animal husbandry. The high prevalence of tetracycline and clindamycin resistance underscores the need for judicious antibiotic use and the development of region-specific treatment guidelines. The identification of novel STs and the comprehensive analysis of virulence factors provide valuable insights for future vaccine development and targeted interventions. Continuous resistance monitoring and surveillance are essential to mitigate the impact of GBS infections on maternal and neonatal health, particularly in regions with high rates of antibiotic resistance. These findings emphasize the importance of integrating molecular epidemiology into public health strategies to combat GBS infections effectively.

## Data Availability

The original contributions presented in the study are publicly available. This data can be found here: https://nmdc.cn/resource/genomics/project/detail/NMDC10019610.
