# Supplementary material for: Molecular epidemiology and antibiotic resistance of group B Streptococcus in pregnant women and neonates from Haikou, China: implications for vaccine development and antimicrobial stewardship
Source: Front Cell Infect Microbiol. 2025 Sep 9;15:1655649. doi: 10.3389/fcimb.2025.1655649 (PMC12454435; doi:10.3389/fcimb.2025.1655649)
Supplement: Supplementary file 2 [file Table2.docx]

Supplementary Table 2: Distributions and relationships among multilocus sequence type, clonal complex, and serotype among the 138 GBS isolates analyzed in this study.

| Clonal Complex (Number of isolates) | Sequence Type (Number of isolates) | Serotype (Number of isolates) |
| --- | --- | --- |
| CC651 (41) | ST862 (27) | III (27) |
|  | ST485 (6) | Ia (6) |
|  | ST314 (3) | Ia (3) |
|  | ST103 (1) | Ia (1) |
|  | ST651 (1) | III (1) |
|  | ST1983 (1) | III (1) |
|  | ST1984 (1) | III (1) |
|  | ST1985 (1) | III (1) |
| CC19 (40) | ST529 (19) | V (19) |
|  | ST19 (14) | V (9), III (5) |
|  | ST27 (3) | III (3) |
|  | ST885 (2) | II (2) |
|  | ST1986 (1) | V (1) |
|  | ST1989 (1) | III (1) |
| CC1 (23) | ST1 (19) | Ib (8), V (7), VI (2), III (1), IX (1) |
|  | ST4 (2) | Ia (2) |
|  | ST1987 (1) | VI (1) |
|  | ST1988 (1) | V (1) |
| CC10 (23) | ST10 (10) | II (6), Ib (4) |
|  | ST12 (10) | Ib (10) |
|  | ST1373 (1) | Ib (1) |
|  | ST1990 (1) | Ib (1) |
|  | ST1991 (1) | II (1) |
| CC23 (6) | ST24 (3) | V (3) |
|  | ST23 (1) | Ia (1) |
|  | ST249 (1) | Ia (1) |
|  | ST890 (1) | V (1) |
| CC17 (5) | ST17 (5) | III (5) |
